# Relationship between equine herpesvirus‐1 viremia and abortion or equine herpesvirus myeloencephalopathy in domesticated horses: A systematic review

**DOI:** 10.1111/jvim.16948

**Published:** 2023-12-09

**Authors:** Gisela Soboll‐Hussey, David C. Dorman, Brandy A. Burgess, Lutz Goehring, Peggy Gross, Claire Neinast, Klaus Osterrieder, Nicola Pusterla, David P. Lunn

**Affiliations:** ^1^ College of Veterinary Medicine Michigan State University, Veterinary Medical Center, Room G331, 784 Wilson Road East Lansing, Michigan 48824 USA; ^2^ College of Veterinary Medicine North Carolina State University, 1060 William Moore Drive Raleigh, North Carolina 27607 USA; ^3^ College of Veterinary Medicine University of Georgia, 2200 College Station Road Athens, Georgia 30602 USA; ^4^ College of Agriculture, Food and Environment, Maxwell H. Gluck Equine Research Center, University of Kentucky, 1400 Nicholasville Road Lexington, Kentucky 40546‐0099 USA; ^5^ Institut für Virologie, Freie Universität Berlin, Robert‐von‐Ostertag‐Strasse 7 Berlin 14163 Germany; ^6^ School of Veterinary Medicine University of California, Davis, One Garrod Drive Davis, California 95616 USA; ^7^ School of Veterinary Science University of Liverpool, Leahurst Campus, Chester High Road Neston CH64 7TE United Kingdom

**Keywords:** abortion, diagnosis, equine, equine herpesvirus myeloencephalopathy, herpesvirus‐1, randomized clinical trial, systematic review, viremia

## Abstract

**Background:**

Equine herpes virus type 1 (EHV‐1) infection in horses is associated with upper respiratory disease, neurological disease, abortions, and neonatal death.

**Objective:**

To determine if there is an association between the level and duration of EHV‐1 viremia and either abortion or equine herpesvirus myeloencephalopathy (EHM) in domesticated horses?

**Methods:**

A systematic review was performed searching numerous databases to identify peer reviewed reports that evaluated viremia and EHM, or viremia and abortion published before January 19, 2021. Randomized controlled trials and observational studies were assessed for risk of bias or publication quality.

**Results:**

A total of 189 unique studies were identified, of which 34 met the inclusion criteria. Thirty studies evaluated viremia and neurologic outcomes including 4 observational studies. Eight experimental studies examined viremia and abortion, which used the Ab4 and OH03 virus strains or recombinant Ab4 derivatives. Incidence rates for both EHM and abortion in experimental studies varied among the studies as did the level of evidence. Viremia was generally detectable before the onset of either EHM or abortion. Risk of bias was generally low to moderate, sample sizes were small, and multiple studies reported negative outcome data.

**Conclusions and Clinical Importance:**

The results of this study support that viremia is regularly present before EHM or abortion occurs. However, no inferences could be made about the relationship between the occurrence of either neurological signs or abortion and the magnitude or duration of viremia.

**Abbreviations**
EHM: equine herpesvirus‐1 myeloencephalopathy
EHV‐1: equine herpesvirus‐1
GRADE: Grading of Recommendations, Assessment, Development, and Evaluation
PICO: Population, Intervention, Comparator, and Outcome
RCTs: randomized clinical trials

## 1 INTRODUCTION

Equine herpesvirus‐1 (EHV‐1) is a *Varicellovirus* in the *Alphaherpesvirinae* subfamily of the *Herpesviridae* and infects horses worldwide.^1^ The clinical manifestations associated with EHV‐1 include respiratory disease, pyrexia, abortion, neonatal death, chorioretinopathy, and a neurologic disease known as equine herpesvirus myeloencephalopathy (EHM).^2^ Infection and transmission of EHV‐1 occurs via the respiratory tract by direct horse‐to‐horse contact, or indirectly from contact with contaminated nasal secretions, aborted fetuses, placenta, and fomites. EHV‐1 infects the respiratory epithelium from which it is transported to regional lymph nodes before establishing a cell‐associated viremia, which is thought to be central in the pathogenesis of abortions and EHM.^1^ During the cell‐associated viremia, EHV‐1 is transported to sites of secondary infection, which include the central nervous system (CNS), the pregnant uterus, and the eye. At these sites, contact between infected leukocytes and the vascular endothelium leads to endothelial cell infection, inflammation, thrombosis and tissue necrosis, and disease outcomes including abortion and EHM.^1^

Abortions following EHV‐1 infection usually occur in the third trimester. Infection of vascular endothelial cells of the endometrium lead to vasculitis, thrombosis, microcotyledonary infarction, perivascular cuffing, and, in some cases, transplacental spread of virus at the sites of vascular lesions.^3^ Thromboischemic necrosis of the cotyledons and intercotyledonary stroma then cause placental detachment and result in the death of the fetus.^4^ Foals born to EHV‐1 infected mares can be affected by a severe pneumonitis and usually succumb to the infection or require euthanasia within days of birth.^5^ Clinical signs associated with EHM are variable in degree and commonly involve some functional neurological abnormality, primarily of the hindquarters of the affected horse.^2^ These abnormalities include hind‐end ataxia, urinary incontinence, and loss of tail muscle tone.^2^ Severely affected horses can become recumbent.^2^ Neuropathologic lesions seen in horses with EHM include vasculitis, axonal degeneration, and thick cuffs of lymphocytes and histiocytes surrounding small blood vessels in the spinal cord and meninges.^2^, ^6^

While there are many factors still unknown about EHV‐1 pathogenesis, a cell‐associated viremia is considered central in the pathogenesis and thought to be a prerequisite for EHV‐1‐induced abortions and EHM.^7^ Furthermore, a positive correlation between the duration and magnitude of viremia and incidence of EHM is suggested.^8^ There is also evidence that more virulent strains of EHV‐1 including Ab4 produce EHM and abortion at higher rates when compared with less virulent such as V592.^2^, ^3^ Finally, a single nucleotide polymorphism at position 2254 in the DNA polymerase gene (ORF 30) is linked with an increased occurrence of EHM.^9^, ^10^, ^11^

Based on this knowledge our panel of experts formulated the following review question: Is there a relationship between either the level or the duration of equine herpesvirus‐1 viremia and either abortion or EHM in domesticated horses? This research question was addressed using systematic review methods. The available data did not support meta‐analyses or other quantitative approaches therefore a qualitative approach to data synthesis was used.

## 2 MATERIALS AND METHODS

### 2.1 Problem formulation and protocol development

A systematic review study protocol was developed using guidelines provided by the Cochrane collaboration.^12^ The protocol detailed the research question, outcomes of interest, outlined a search strategy and the process of data extraction and provided criteria for rating the quality of evidence (Table S1). The specific review question and Population, Intervention, Comparator, and Outcome (PICO) statement for the systematic review were as follows.

#### 2.1.1 Review question

Is there a relationship between the level and duration of equine herpesvirus‐1 viremia and either abortion or EHM in domesticated horses? The review question was developed and refined through a series of problem formulation steps including preliminary literature searches. This systematic review utilized data collected from previous studies. Therefore, ethical approval was not required.

#### 2.1.2 PICO statement

The following PICO (problem/population, intervention, comparison, and outcome) framework was developed:

- Population: Domesticated equids without sex, age, or breed restrictions.
- Intervention/Exposure: Equids experimentally infected or naturally exposed to EHV‐1 infection.
- Comparator: Measurement/detection of viremia and association with severity of clinical, clinico‐pathological, and pathological signs of abortion, neonatal loss, or EHM.
- Outcome: All clinical outcomes that reflect symptomatic EHV‐1 infection in horses with abortion, neonatal loss, or EHM. Presence and degree of viremia.

#### 2.1.3 Inclusion and exclusion criteria

The following inclusion and exclusion criteria were used.

Inclusion criteria:

- Domesticated equids without age, breed, or immunological status restriction.
- Any experimental challenge or natural infection with measurement of disease and of viremia.
- Study included clinical outcomes that reflect symptomatic EHV‐1 infection resulting in either abortion, neonatal death or EHM. Main outcomes include abortion or neonatal loss (1‐week‐old foal or younger) and neurologic signs suggestive of EHM.
- Studies were not excluded on the basis of year of publication, language, or quality.

Exclusion criteria (reason was recorded):

- Absence of an EHV‐1 challenge trial or exposure.
- Absence of the selected clinical or virological outcomes.
- Wrong species of virus.
- Wrong species (not equine).
- Purely descriptive observational studies.
- No original data.

### 2.2 Search strategy

The review team initially considered existing systematic reviews to address or help to address its research question. English‐language systematic reviews conducted within the last 5 years were sought using searches in PubMed, PROSPERO (CRD), and CAMARADES. No relevant systematic reviews on this topic were identified.

In addition to consideration of systematic reviews, a search for bibliographic references was performed through PubMed, Web of Science, Cochrane, CAB Abstracts, AGRICOLA, Global Index Medicus regional databases to include African Index Medicus (AIM), Eastern Mediterranean Region (IMEMR), South‐East Asia Region (IMSEAR), Latin America and the Caribbean Literature on Health Sciences (LILACS), Western Pacific Region Index Medicus (WPRO) to locate studies. The search was limited to domesticated horses and performed without sex, age, breed, or language restrictions. Only peer‐reviewed publications were considered. The search strategies included descriptors or words in the text related to abortion, foal death, EHM, and viremia. The search was developed with input from a librarian (*Peggy Gross*) with expertise in the conduct of systematic reviews (Table S1). The initial literature search was performed on December 20, 2019 and the original set of citations were uploaded into Covidence. A last update on available citations was done on January 19, 2021.

### 2.3 Study selection

Screening and quality assessment were tracked in Covidence (www.covidence.org). The evaluation of titles, abstracts, and the full text were independently performed by a team of 2 reviewers at either the initial screening (Nicola Pusterla, Klaus Osterrieder) or full text review (David Dorman, Claire Neinast) steps. Disagreements were resolved by either discussion or when consensus could not be reached using a third reviewer. A list of excluded studies in the full text screening stage, with the reason for exclusion, is provided in Table S2.

#### 2.3.1 Data extraction

Extraction of originally published graphical data relied on DigitizeIt version 2.5.1. (Braunschweig, Germany). Data were extracted from included studies by 1 member of the review team (Dave Dorman) and checked by a second member (Claire Neinast) for completeness and accuracy. Any discrepancies in data extraction were resolved through discussion. The extracted data were used to summarize study designs and findings and/or to conduct post‐hoc statistical analyses (Table S1). Specific study endpoints that were extracted included: demographic data, virus challenge protocols including virus strain and dose, clinical signs, reproductive tract or foal pathology, neuropathology, and virology data including the presence or absence of viremia, duration and quantification of viremia, and methods used.

#### 2.3.2 Risk of bias evaluation

The risk of bias domains and questions for assessing risk of bias in experimental studies were based on established guidance for animal studies.^13^ The following domains were assessed: blinding of participants and personnel, random selection of animals for outcome assessment, blinding of outcome assessment, incomplete outcome data, selective reporting, and other bias. Experimental studies were independently assessed by 2 assessors (David Dorman, Claire Neinast) who answered all applicable risk of bias questions with 1 of 3 options (low risk of bias, unclear risk of bias, or high risk of bias) following prespecified criteria (Table S1). Any discrepancies were resolved through discussion or the use of a third individual. Risk of bias was assessed at the outcome level. Assessment of the quality of observational studies was performed using the Joanna Briggs Institute (JBI) Critical Appraisal Checklist for Case Reports (last amended in 2017).^14^ This tool assesses whether the following components are clearly described: demographic characteristics, history, clinical, diagnostic tests or assessment methods, intervention, post‐intervention clinical condition, adverse events and whether the case report provides takeaway lessons. Observational studies were independently assessed by 2 assessors who used 1 of 4 options (yes, no, unclear, not applicable) for each criterion. All assessments were performed by individuals that did not participate in the original research study.

#### 2.3.3 Strategy of data synthesis

A narrative synthesis (eg, study design, year of publication, subject baseline demographics, sample size, country where study was conducted, interventions, and the results from each study) was performed for each outcome (abortion or neurologic effects).

## 3 RESULTS

### 3.1 Results of the search

**FIGURE 1:**
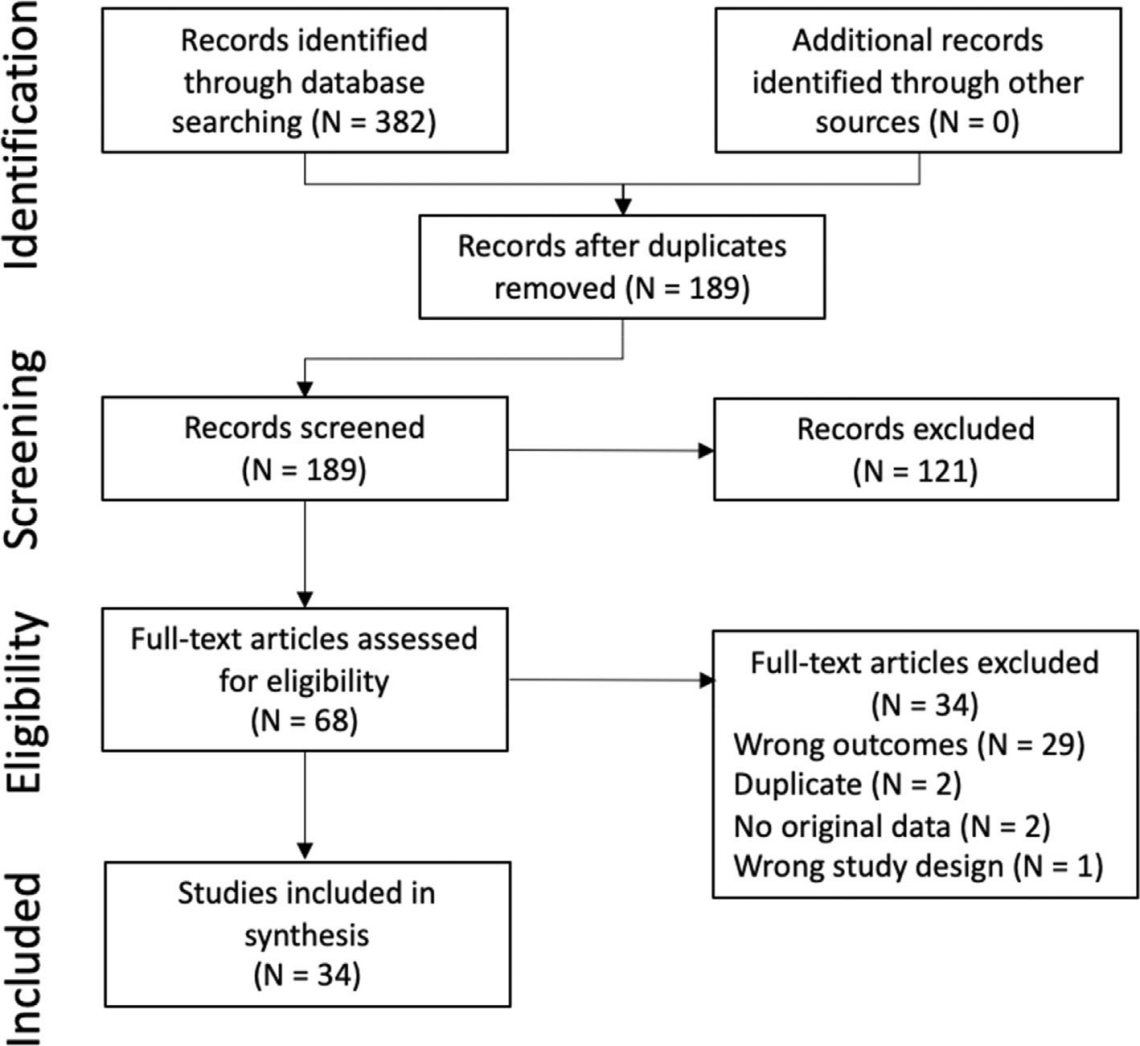
PRISMA flowchart for the literature search process.

The search strategy identified 382 citations, of which 193 were duplicate citations. Another 121 citations were excluded based on the title or abstract. Literature was almost entirely identified and retrieved from electronic bibliographic sources. No studies were identified from hand searching reference lists provided in the studies that met inclusion criteria. A total of 68 studies were assessed for inclusion using a review of the full text. Thirty‐four studies met the inclusion criteria for this review. A flow diagram for inclusion of studies in the systematic review is provided in Figure 1.

### 3.2 Description of the included studies

A total of 34 studies met our inclusion criteria. The key characteristics of these studies are summarized in Table 1. Of the 34 studies, 30 evaluated viremia and neurologic outcomes (Table 2). The most used strains of virus in the experimental studies were Ab4 (17 studies),^3^, ^10^, ^15^, ^16^, ^17^, ^18^, ^19^, ^20^, ^21^, ^22^, ^23^, ^24^, ^25^, ^26^, ^27^, ^28^, ^29^ Ab4 mutants including strains lacking an open reading frame (7 studies),^10^, ^19^, ^20^, ^23^, ^25^, ^26^, ^28^ and OH03 (7 studies).^3^, ^8^, ^17^, ^30^, ^31^, ^32^, ^33^ The remaining studies used Army 183, 03P37, 970P70 or FR‐56628.^21^, ^34^, ^35^, ^36^, ^37^ Seven studies compared the virulence of 2 or more strains.^3^, ^8^, ^16^, ^17^, ^21^, ^30^, ^35^ Some studies evaluated vaccine or treatment efficacy in horses inoculated with EHV‐1. Incidence rates for EHM in experimental studies varied among the different studies. Overall, the incidence rate of neurologic signs including ataxia and quadriplegia was approximately 13% (n = 474). This incidence rate does not include the occurrence of less specific signs like anorexia or CNS depression. Findings between studies varied with some showing evidence for a correlation between levels of viremia and development of EHM,^8^, ^33^ some indicating that the duration of viremia may be more important for predicting EHM,^32^ and some studies finding no correlation between level or duration of viremia and incidence of EHM.^15^, ^19^, ^24^, ^27^ Data combining the findings from all neurological horses seem to suggest that an association (*P* = .01) between duration of viremia is more important for the risk of ataxia than the number of infected PBMCs, that is, the level of viremia. However, data analysis was complicated by the fact that often no individual viremia data was reported, assays for measuring viremia differed, and the overall incidence for horses exhibiting neurological signs in many studies was low as were numbers of horses per experimental group.

**TABLE 1:** Selected demographic characteristics of study populations.

Citation no.	Study	Breed	Total number of horses	Sex	Age	Pre‐study EHV‐1 status	Treatments	Comments
8	Allen (2008)	Thoroughbred (n = 25) Mixed breed (n = 11)	36	F	4 to 28 y	Unvaccinated during year before study	>20 y of age: n = 12 inoculated with EHV‐1 (T953), n = 12 inoculated with EHV‐1 (T262). <15 y of age inoculated with EHV‐1 (T953), n = 12.	Experimental trial.
30	Allen and Breathnach (2006)	Mixed breed derived from single Standardbred sire	20	F (n = NR), M (n = NR)	7 to 8 mo	Seronegative 1 wk before study	Ten EHV‐1 isolates were used for foal inoculations—five isolates were considered neuropathogenic (n = 10) five were abortigenic (n = 10).	Experimental trial. Data pooled for each viral class.
15	Brosnahan et al. (2010)	Multiple breeds	14	CM (n = 8); F (n = 6)	2 to 18 y	Negative antibody titer (SN ≤ 1:24)	Horses treated with either sigB3/siOri2 siRNA (n = 10) or siLuc control group (n = 4).	
42	Burki et al. (1990)	Haflinger (n = 6) Shetland Pony (n = 3) Thoroughbred (n = 1)	10	PF (n = 10)	3‐12 y	Negative serological titers in sentinel horses during year before experiment	Horses vaccinated with either a live or inactivated vaccine. Challenged 3 wk after last vaccination.	Only data for PF are included in this review.
16	Edington et al. (1986)	Welsh Mtn Ponies	8	F	6 to >18 y	NR	Mares inoculated with AB4 yearlings with EHV‐1 strain 2252.	Two experiments were performed.
		Welsh Mtn Ponies	5	F (n = 3) M (n = 2)	Yearlings			
38	Estell et al. (2015)	Quarter Horse (n = 5) Standardbred (n = 1) Warmblood (n = 1)	7	F (n = 4) MC (n = 1) M (n = 2)	3 to 15 y	Previous exposure (via known outbreak or exposure to new additions at boarding facility)	5/7 horses were treated (eg, flunixin meglumine, steroids, valacyclovir) before referral.	Retrospective case series.
3	Gardiner et al. (2012)	Ponies (NOS)	11	F (n = 4) PF (n = 7)	3 y	SN titer < 1:32	Ponies were inoculated with either OH03 or Ab4.	Two experiments were performed. Mares inoculated at approximately 270‐290 d of gestation.
			9	F (n = 2) PF (n = 7)				
43	Gleeson and Coggins (1980)	Welsh Mtn Ponies (n = 17) Standardbred (n = 4)	21	PF	NR	Unvaccinated during year before study, isolated herd for several years with no known epizootic EHV1 infection	Horses were inoculated with either Army 183 or KyB.	The two studies were conducted over two seasons.
31	Goehring et al. (2010a)	Mixed‐breed pony	24	F (n = 12) M (n = 12)	11‐13 mo	Negative SN titers	Ponies were vaccinated 3 times and challenge infected on Day 121 of the experiment	
34	Goehring et al. (2010b)	Standardbred	4	F	14 to 20 y	Negative SN titer (between 1:128 and 1:256), negative glycoprotein G Ab (ELISA), negative CF titer (≤1:10)		Data from two unexposed controls not included.
17	Goehring et al. (2013)	Horses (Western stock)	8	Mixed	Yearling	SN titer ≤ 1:32	Yearlings were inoculated with Ab4 and aged horses were inoculated with OH03.	Two separate experiments were performed with yearlings. Aged horses were part of an antiviral drug study.
		Ponies (NOS)	10					
		Horses (NOS)	10	NR	>18 y	Negative Ab (EHV‐1 specific glycoprotein G) ELISA (<0.5 absorbance)		
32	Goodman et al. (2006)	Mixed‐breed (NOS)	15	F	3‐10 y	Negative EHV1 neutralization titers (SN < 1:24)	Horses (n = 5/group) were allocated to control, inactivated, and modified‐live virus vaccine treatment groups. Challenge occurred 59 d after the initial vaccination.	
35	Gryspeerdt et al. (2010)	Shetland Pony	12	M	6 mo to 2 y	Negative SN titer (<2), negative IPMA (<10)	Two viral strains used (03P37, 97P70).	Two horses euthanized for tissue virology studies at 1 dpi before onset of fever
18	Heldens et al. (2001)	Welsh Mtn Ponies	9	PF	3 y	Positive CF and VN antibodies to EHV1 or EHV 4	Vaccine trial using an inactivated vaccine at 5, 7‐9 mo of gestation and challenged 4 wk after last vaccination.	Data from foals not included in the review.
19	Holz et al. (2017)	Horses (NOS)	25	Mixed	Yearling	SN titer < 4 (EHV1) SN titer < 40 (EHV4)	Multiple viral strains used (Ab4, Ab4 N752, b4 gD4)	Multiple viral strains used (Ab4, Ab4 N752, b4 gD4)
20	Hussey et al. (2013)	Ponies (NOS)	10	NR	NR	SN titer < 1:32		Three experimental conditions using different virus strains.
			24	NR	9‐18 mo			
44	Kydd et al. (2003)	Ponies (NOS)	14	PF	3 to 20 y	Negative CF antibody titer (1 exception—elevated CF titer present)	Some mares were vaccinated with an inactivated vaccine. Mares previously infected with EHV‐1 were challenged 4 wk after last vaccination.	Third group (multiply infected horses) not included in the review.
33	Maxwell et al. (2017)	Light horse breeds (NOS)	12	F	>20 y	Seronegative for anti‐EHV‐1 antibodies (ELISA)	Therapeutic trial with horses given placebo or prophylactic valacyclovir for 1 or 2 wk.	Data from horses given valacyclovir after detection of fever not included.
22	Mumford et al. (1991)	Ponies (NOS)	19	PF	NR	Unknown	Vaccinated with immune stimulating complexes. Challenge occurred 3 wk after last vaccination.	Two experimental groups: control and mares
21	Mumford et al. (1994)	Ponies (NOS)	62	PF (n = 61) F (n = 1)	NR	Isolated and monitored (clinically and serologically) 6 mo before challenge infection. Pre‐challenge VN antibody titers provided for some cohorts (range 10^<0.6^ to 10^2.2^)	Multiple viruses and doses used.	Data for different doses were pooled.
45	Patel et al. (2003)	Welsh Mtn Ponies	18	PF	NR	No or low virus neutralizing antibody to EHV‐1 or EHV‐4.	Some mares (n = 6/group) were vaccinated at 3.4‐4.1 mo of gestation or between 5.4 and 5.8 mo of gestation. Challenged between 8.4 and 8.9 mo pregnant.	
24	Perkins et al. (2013)	Horses (NOS)	13	CM (n = 6) F (n = 7)	3‐20 y	Negative antibody titer (SN ≤ 64). Unknown vaccination history	Trials with small interfering RNAs (siRNAs) or an irrelevant siRNA administered before and after infection.	
23	Perkins et al. (2019)	Icelandic	15	CM (n = 10), F (n = 5)	2.5 y	EHV‐1 naïve herd.	Horses were initially uninfected (control) or exposed to EHV‐1 (Ab4 or Ab4ΔORF1/71) and then challenged 6 mo later.	Data from second challenge extracted.
41	Pusterla et al. (2008)	Thoroughbred (n = 41) NR (n = 27)	68	NR	Adult (NOS)	Involved in a reported EHV‐1 outbreak (natural exposure).		Three cohorts of horses: febrile (n = 12), neurologic (n = 15), and subclinical (n = 41).
40	Pusterla et al. (2012)	Quarter horse (n = 32), draft breeds (n = 30), standardbred (n = 15), others (n = 5)	82	F (n = 34) CM (n = 48)	3 to 30 y	Natural exposure at packing station. Unvaccinated during year before outbreak.		Data from mules not included.
39	Pusterla et al. (2021)	Warmblood (n = 24), others (n = 7)	31	F (n = 17) CM (n = 14)	1 to 27 y	Natural infection at performance farm discovered during routine dental care and vaccination	Horses treated with valacyclovir (n = 31), flunixin meglumine (n = 26) and/or heparin (n = 26).	
26	Schnabel et al. (2018)	Icelandic	16	F (n = 8) CM (n = 8)	2 to 4 y	Previously infected with EHV‐1 (NY03) at 7 mo. Protective immunity had “waned to values typically observed in EHV‐1 susceptible horses”	Used two viruses (Ab4 or Ab4ΔORF2).	Data from uninfected control group are not included
25	Schnabel et al. (2019)	Icelandic	24	F (12) M (12)	3‐5 y	EHV‐1 naïve herd.	Horses were initially uninfected (control) or exposed to EHV‐1 (Ab4 or Ab4ΔORF1/71) and then challenged 9 mo later.	Data from second challenge extracted.
36	Soboll et al. (2010)	Ponies (NOS)	26	NR	2‐7 y	Previously exposed to EHV‐1 > 12 mo ago	Ponies were challenge infected 8 wk after the last vaccination.	Ponies expressing different MHC I haplotypes were used as well as a recombinant modified vaccine and unvaccinated controls. Clinical scores were derived by allocating 1 point for cough, ocular discharge, nasal discharge, depression, and pyrexia (>38.6°C) and calculating the sum.
37	Sutton et al. (2020)	Welsh Mtn Ponies	4	M	10 mo	Seronegative for EHV‐1 and EHV‐4, SN and CF assay, no history of EHV infection		Experimental trial—no comparator group or dose‐response data (virulence study).
10	Van de Walle et al. (2009)	NR	9	NR	NR	SN antibody titer < 24	Two Ab4 strains used (parental rNY03_N752 of mutant rNY03_D752).	Viremia a score of 0: no virus isolated, score = 1:1‐10 plaques using 5 × 10^6^ PBMCs, score = 4: plaques seen using 5 × 10^3^ PBMCs.
27	Wilson et al. (2019)	NR	8	Mixed (NOS)	Yearling	Unvaccinated, EHV‐1 titers ≤ 1:2, EHV‐4 titers ≤ 1:20		Data from controls (uninfected) not included. Total of 11 horses used, staggered start with some controls were exposed to EHV‐1 (n = 8).
28	Wimer et al. (2018)	Icelandic ponies	15	F (n = 5) CM (n = 10)	2.5 y	Naïve herd	Groups included uninfected (control) or exposed to EHV‐1 (Ab4 or Ab4ΔORF1/71).	Data from uninfected controls (n = 5) are not included in the review.
29	Zarski et al. (2021)	Mixed breeds (NOS)	7	M (n = 5) F (n = 2)	2 y	SN antibody titer ≤ 1:8 for EHV‐1	Horses received (IN) either a human adenovirus vector expressing the EHV‐1 IR2 protein or a null adenovirus vector 2 d before EHV‐1 challenge.	Data were pooled for these groups.

Abbreviations: CF, complement fixing; CM, castrated male (gelding); F, female; IN, intranasal; IPMA, immunoperoxidase monolayer assay; M, male; mo, month; Mtn, mountain; NOS, not otherwise specified; NR, not reported; PF, pregnant female; SN, serum neutralization; VN, virus neutralizing; y, year.

**TABLE 2:** Summary of main findings from studies evaluating neurologic effects following EHV‐1 exposure.

Study identifier	Study design	Virus	Dose (route)	Outcome incidence	Main findings: viremia
Allen (2008)	RCT	Neuropathogenic: T953 (OH03)	10^7^ PFU (IN)	Neurologic signs: 9/24	Mean peak viral load (pcr relative quantification) asymptomatic horses = 52.9 ± 99.8; neurologic horses = 3740 ± 5600 (*P* = .02).
		Abortogenic: T262	10^7^ PFU (IN)	Neurologic signs: 0/12	Mean peak viral load non‐neuropathogenic strain (Day 6) = 7.1 ± 23.7.
Allen and Breathnach (2006)	NRES	Abortogenic: T61, T75, T220, T480, T677	10^7^ PFU (IN)	Fever (>39°C): 10/10, nasal shedding: 10/10, duration of nasal shedding 4‐7 d, neurologic signs: 0/10.	Mean peak viral load: Day 9 = 29.5 ± 37.9. Viremia duration = 6 d.
		Neuropathogenic: T313, T510, T672, T935, T946	10^7^ PFU (IN)	Fever (>39°C): 10/10, nasal shedding: 10/10, duration of nasal shedding 5‐12 d, neurologic signs: 2/10; 1/2 (T672); 1/2 (T935).	Mean peak viral load (pcr relative quantification): Day 7 = 165 ± 170. Viremia duration = 12 d. Duration of nasal shedding and viremia were greater in foals inoculated with neuropathogenic isolates.
Brosnahan (2010)	RCT	Ab4 siLuc [controls]	10^7^ PFU (IN)	Nasal discharge: 4/4, fever > 38.5°C: 4/4, neurologic signs: 3/4.	Viremia (qpcr) data not reported for neurologic and non‐neurological horses. Viral copies in CSF from neurologic horses had 0 to 1.6 × 10^5^ genome copies/mL (mean 5.9 × 10^4^ ± 7.5 × 10^4^. Viral copies in spinal cord from neurologic horses had 0.7 to 3.0 × 10^6^ genome copies/mL.
		Ab4 sigB3/siOri2		Nasal discharge: 10/10, fever > 38.5°C: 10/10, neurologic signs: 2/10.	
Eddington (1986)	NRES	Ab4	10^5.5^ TCID_50_ (IN) and 1.5 × 10^5.5^ TCID_50_ (SQ)	Fever ≥ 38.5°C: 8/8, neurologic signs: 3/8. CNS arteriolar and capillary thrombi unknown incidence. CNS hemorrhages from 2 ataxic mares were associated with extensive endothelial cell fluorescence and thrombus formation.	Viremia (equine kidney cell culture): 8/8, viremia from 3 to 10 dpi. Nasal shedding (8/8), duration from 1 to 10 d. Recovery of virus from CNS (1/8).
		2252	10^5.5^ TCID50 (IN)	Fever ≥ 38.5°C: 2/5, neurologic signs: 0/5.	Viremia (equine kidney cell culture): 0/5. Nasal shedding (5/5) duration from 4 to 7 d.
Estell (2015)	Retrospective case series	Genotype D_752_	Unknown	Fever: 4/7, neurologic signs: 7/7, case fatality: 2/7. Nonsurvivors with neurologic signs had focal CNS hemorrhages, multifocal vasculitis of small arterioles, nonsuppurative meningoencephalomyelitis, and multifocal CNS malacic lesions associated with vasculitis.	Viremia: 7/7. Peak viral load in blood was higher in nonsurvivors (2.05 × 10^4^ and 1.02 × 10^5^) vs survivors (143‐4340; median 3150 gB gene copies/million cells).
Gardiner (2012)	NRES	OHO3	5 × 10^7^ PFU (IN)	Fever ≥ 1°C rise above baseline: 7/7, neurologic signs: 1/7	Viremia (pcr): 7/7. Viremic between Days 5 and 8 post‐inoculation, with a duration of 1‐4 d. Mare with neurologic signs: peak viremia (6 dpi): 10^3.4^; no abortion (5‐7 dpi): 10^2.9^‐10^3.6^ EHV‐1 gB copies/10^6^ beta β copies
		Ab4		Fever ≥ 1°C rise above baseline: 7/7, neurologic signs: 0/7	Viremia (pcr): 7/7. Viremic between Days 5 and 8 with a duration of 1‐3 d. Peak viremia (5‐9 dpi): 10^2.3^‐10^3.2^ EHV‐1 gB copies/10^6^ β Actin copies
Goehring (2010a)	RCT	OH03	5 × 10^7^ PFU (IN)	Fever ≥ 38.6°C: 24/24, neurologic signs: 0/24	Viremia (pcr) control group: Peak mean (8 dpi) = 10^2.9^ EHV‐1 gB copies/10^6^ beta Actin copies. Viremia (pcr) vaccinated groups: Peak mean (6 dpi) = 10^1.6^ EHV‐1 gB copies/10^6^ β Actin copies.
Goehring (2010b)	NRES	Neuropathogenic EHV‐1 strain	5 × 10^7.6^ PFU (NP)	Fever ≥ 38.5°C: 4/4, mild neurologic signs: 4/4, neuropathology 1/4.	Viremia (pcr): 1/4, Peak viremia (12 dpi) = 1.5 × 10^5^ DNA copies/2.5 × 10^6^ PBMC
Goehring (2013)	NRES	Ab4	5 × 10^7^ PFU (NP)	Fever: 8/8, neurologic signs: 1/8	Viremia (pcr): 8/8. Viremia duration = 2‐6 d with a median = 3.5 d; 0‐7 d duration with a median of 2.5 d in group 2, and for 0‐8 d with a median of 4 d in group 3.
		Ab4		Fever: 8/10, neurologic signs: 0/10	Viremia (pcr): 8/10. Viremia duration = 0‐7 d median = 2.5 d.
		OH03	1 × 10^7^ PFU (NP)	Fever: 8/10, neurologic signs: 6/8	Viremia (pcr): 9/10. Viremia duration = 0‐8 d median = 4 d.
Goodman (2006)	RCT	OHO3 Controls	5 × 10^6^ PFU (NP)	Fever ≥ 38.5°C: 5/5, nasal shedding: 5/5; duration of nasal shedding: 3.8 d, neurologic signs: 3/5.	Viremia (qpcr): 5/5. Peak mean viremia (9 dpi) 1.5 × 10^5^ genome copies per 10^9^ 18S rRNA gene copies.
		OHO3 MLV vaccine		Fever ≥ 38.5°C: 5/5, nasal shedding 1/5, duration of nasal shedding: 0.2 d, neurologic signs: 0/5.	Viremia (qpcr): 5/5. Peak mean viremia (9 dpi) 3.3 × 10^5^ genome copies per 10^9^ 18S rRNA gene copies.
		OHO3 Inactivated vaccine		Fever ≥ 38.5°C: 5/5, nasal shedding controls: 5/5, duration of nasal shedding: 3.2 d, neurologic signs: controls: 3/5.	Viremia (qpcr): 5/5. Peak mean viremia (9 dpi) 8.1 × 10^4^ genome copies per 10^9^ 18S rRNA gene copies.
Gryspeerdt (2010)	NRES	03P37 (neuropathic)	10^6.5^ TCID_50_ (50:50: IN/PO)	Fever ≥ 38.5°C: 5/5, nasal shedding 5/6, neurologic signs: 0/5.	Viremia (plaque assay): 4/5. Quantity of viremia was variable between animals (n = 2‐3) after 3 dpi. At 3 dpi mean number of plaques/10^7^ PBMC = 3.3. Maximum = 36 plaques/10^7^ PBMC (4 dpi, n = 1).
		97P70 (nonneuropathic)		Fever ≥ 38.5°C: 5/5, nasal shedding 6/6, neurologic signs: 0/5.	Viremia (plaque assay): 5/5. Quantity of viremia was variable between animals (n = 2‐3) after 3 dpi. At 3 dpi mean number of plaques/10^7^ PBMC = 1.0. Maximum = 42 plaques/10^7^ PBMC (4 dpi, n = 1).
Heldens (2001)	NRES	Ab4 Controls	2 × 10^6.0^ TCID_50_ (IN)	Fever (>38.8°C): 4/5; nasal shedding: 5/5, neurologic signs (ataxia): 1/5	Viremia (horse kidney cells): controls: 5/5; viral titers (PBMC) not provided, mean duration of viremia: 4.3 d.
		Ab4 Vaccinates		Fever (>38.8°C): 2/4; nasal shedding: 5/5, neurologic signs (ataxia): 0/5	Viremia (horse kidney cells): 5/5, viral titers (PBMC) not provided, mean duration of viremia: 4.0 d.
Holz (2017)	NRES	Ab4	5 × 10^7^ PFU (IN)	Fever (>38.6°C) 8/8; neurologic signs: Ab4: 3/8.	Viremia (pcr): 8/8. Peak mean viremia (pcr): 6 Dpi: 699 ± 751 Gb copy number/10^5^ β‐Actin copies. Ab4‐infected horses had higher viremia vs Ab4 N752‐infected horses (*P* < .001) and approached significance when compared to Ab4 gD4‐infected horses (*P* = .07). Duration of viremia = 3 d.
		Ab4 N752 (ORF mutant)		Fever (>38.6°C) 9/9; neurologic signs: 0/9	Viremia (pcr): 9/9. Peak mean viremia (pcr): 7 dpi: 68 ± 78 Gb copy number/10^5^ β‐Actin copies
		Ab4 gD4		Fever (>38.6°C): 8/8. neurologic signs: 0/8	Viremia (pcr): 8/8. Peak mean viremia (pcr): 50 ± 142 Gb copy number/10^5^ β‐Actin copies
Hussey (2013)	NRES	Ab4	5 × 10^7^ PFU (IN)	Fever: 10/10, neurologic signs: 0/10	Viremia (plaque assay): 10/10. Peak median viremia (experiment 3) Ab4 (6 dpi): 2.0 × 10^3^ log gB copies/10^6^ β‐Actin copies. Viremic for 1‐7 d between 3 and 9 dpi.
		Ab4Δ75‐LacZ	5 × 10^7^ PFU (NP aerosol)	Fever: 12/12, neurologic signs: 1/12	Viremia (pcr): 12/12. Viremic for 1‐7 d between 3 and 9 dpi.
		Ab4 or Ab4GFP		Fever: 12/12, neurologic signs: 0/12	Viremia (pcr): 11/12. Peak median viremia (7 dpi): 4.9 × 10^3^ log gB copies/10^6^ β‐Actin copies. Viremic for 1‐7 d between 3 and 9 dpi.
Maxwell (2017)	RCT	T953 (OH03) Controls	10^7^ PFU (IN)	Fever: 6/6, ataxia 4/6, neuropathology: negative.	Viremia (pcr): 6/6. Peak viremia at 9 dpi: mean = 23 000 copies/10^6^ cells.
		T953 (OH03) Prophylactic valacyclovir (1 wk)		Fever: 4/6, ataxia: 1/3, neuropathology: negative.	Viremia (pcr): 3/3, nasal shedding 3/3. Peak viremia at 8 dpi: mean = 960 copies/10^6^ cells.
		T953 (OH03) Prophylactic valacyclovir (2 wk)		Fever: 4/6, ataxia: 0/3, neuropathology: negative.	Viremia (pcr): 3/3, nasal shedding 3/3. Peak viremia at 9 dpi: mean = 59 copies/10^6^ cells.
Mumford (1991)	NRES	AB4 Controls	10^7^ TCID_50_ (IN)	Fever: 9/9, neurologic signs (quadriplegia): 1/9	Viremia (rabbit kidney cells): 9/9
		AB4 Vaccinated		Fever: 10/10, neurologic signs (quadriplegia): 0/10	Viremia (rabbit kidney cells): 10/10
Mumford (1994)	NRES	V592	30 or 50 × 10^7.5^ TCID_50_ (IN aerosol)	Fever: 8/10, neurologic signs (quadriplegia): 0/10	Viremia (rabbit kidney cells): 10/10, mean duration of viremia: 2.5‐3.5 d.
		Ab4	20 × 10^6.7^ TCID_50_ (IN aerosol)	Fever: 8/8, neurologic signs (quadriplegia): 0/8	Viremia (rabbit kidney cells): 8/18, mean duration of viremia: 3.1 d.
			30 × 10^7.5^ TCID_50_ (IN aerosol)	Fever: 7/7, neurologic signs (quadriplegia): 2/7	Viremia (rabbit kidney cells): 7/7, mean duration of viremia: 8.2 d.
			10^3^ to 10^7^ TCID_50_ (IN)	Fever: 35/41, neurologic signs (quadriplegia): 3/41	Viremia (rabbit kidney cells): 15/15
Perkins (2013)	RCT	Ab4 siRNA	10^7^ PFU (IN)	Fever: 7/7, nasal shedding: 7/7. Neurologic signs with histologic evidence of lymphocytic perivascular cuffs in CNS: 3/7	Viremia (qpcr): 7/7, maximum genome copies/10^6^ cells: 18 700 (606‐55 600)
		Ab4 siLuc control		Fever: 6/6, nasal shedding: 6/6. Neurologic signs with histologic evidence of lymphocytic perivascular cuffs in CNS: 2/6	Viremia (qpcr): 6/6, maximum genome copies/10^6^ cells: 7735 (1510‐45 100)
Perkins (2019)	RCT	Null/Ab4	10^7^ PFU (IN)	Fever: 5/5, nasal shedding: 5/5, neurologic signs: 0/5	Viremia (qpcr): 5/5; peak viremia at 5 dpi: 32.1 (31.2‐32.6)
		Ab4/Ab4		Fever: 0/5, neurologic signs: 0/5	Viremia (qpcr): 0/5, peak viremia at 5 dpi: not detected
		Ab4ΔORF1/71/Ab4		Fever: 0/5, neurologic signs: 0/5	Viremia (qpcr): 0/5, peak viremia at 5 dpi: not detected
Pusterla (2008)	Retrospective case series	Febrile	Unknown	Fever: 12/12, nasal shedding: 12/12, neurologic signs: 0/12	Viremia (qpcr): 12/12, mean viral load blood: 1.7 × 10^4^ gene copies/10^6^ cells
		Neurologic		Fever unknown, nasal shedding: 15/15, neurologic signs: 15/15	Viremia (qpcr): 9/15, mean viral load blood: 1.6 × 10^2^ gene copies/10^6^ cells
		Subclinical		Fever 0/41, nasal shedding: 40/41, neurologic signs: 0/41	Viremia (qpcr): 5/41, mean viral load blood: 4.5 × 10^1^ gene copies/10^6^ cells
Pusterla (2012)	Retrospective case series	Asymptomatic (n = 27)	Unknown	Neurologic signs: 0/27	Viremia (qpcr): 27/27, mean viral load blood: 157 ± 30 gene copies/10^6^ cells.
		Neurologic (n = 6)		Neurologic signs: 6/6	Viremia (qpcr): 6/6, mean viral load blood: 2820 ± 740 gene copies/10^6^ cells.
Pusterla (2021)	Retrospective case series	Natural infection	Unknown	Fever: 26/31, nasal shedding: 26/31, neurologic signs: 4/31.	Viremia (qpcr): 13/41, neurologic horses 4/4. Median viral load blood (Day 0 of outbreak) = 6269 [range = 572 to 2.1 × 10^5^] gene copies/10^6^ cells (n = 13).
Schnabel (2018)	RCT	Ab4	10^7^ PFU (IN)	Fever: 8/8, nasal shedding: 8/8, mild ataxia: 1/8	Viremia (qpcr): 8/8, mean viral load blood (6 dpi): 32.3 ± 1.1 Ct values for the gB gene.
		Ab4Δ ORF2		Fever: 8/8, nasal shedding: 8/8, mild ataxia: 1/8	Viremia (qpcr): 8/8, mean viral load blood (6 dpi): 33.4 ± 1.1 Ct values for the gB gen. On d8 pi, higher amounts of viral DNA were detected in PBMC of the Ab4ΔORF2 group vs Ab4 group (*P* < .01)
Schnabel (2019)	RCT	Null/Ab4	10^7^ PFU (IN)	Fever: 8/8, nasal shedding: 8/8, mild ataxia: 1/8	Viremia (qpcr): 8/8; peak viremia at 4‐8 dpi: mean (n = 8): 32.3 ± 1.6 Ct values for the gB gene.
		Ab4/Ab4		Fever: 0/8, nasal shedding: 3/8, neurologic signs: 0/8	Viremia (qpcr): 3/8, peak viremia at 6‐8 dpi: mean (n = 3): 34.3 ± 3.6 Ct values for the gB gene.
		Ab4ΔORF2/Ab4		Fever: 1/8, nasal shedding: 1/8, neurologic signs: 0/8	Viremia (qpcr): 0/5, peak viremia at 8 dpi (n = 1): 37.0 Ct values for the gB gene.
Soboll (2010)	NRES	Army 183 A3/B2 vacc	5 × 10^7^ PFU (IN)	Mean maximum clinical score (1 dpi, n = 10): 0.9, nasal shedding: 10/10, neurologic signs: 0/10.	Viremia (qpcr): 1/10, Mean peak viremia at 8 dpi (n = 10): 0.4 Log gB copy numbers/10^6^ copies of β‐Actin.
		Army 183 A3‐non‐B2 vacc		Mean maximum clinical score (10 dpi, n = 5): 2.4, nasal shedding: 5/5, neurologic signs: 0/5.	Viremia (qpcr): 5/5, Mean peak viremia at 9 dpi (n = 5): 2.8 Log gB copy numbers/10^6^ copies of β‐Actin.
		Army 183 Non‐A3 vacc		Mean maximum clinical score (6 dpi, n = 5): 2.4, nasal shedding: 5/5, neurologic signs: 0/5.	Viremia (qpcr): 3/6, Mean peak viremia at 8 dpi (n = 6): 1.2 Log gB copy numbers/10^6^ copies of β‐Actin.
		Army 183 Control		Mean maximum clinical score (4 dpi, n = 6): 1.5, nasal shedding: 6/6, neurologic signs: 0/6.	Viremia (qpcr): 5/5, Mean peak viremia at 8 dpi (n = 5): 2.3 Log gB copy numbers/10^6^ copies of β‐Actin.
Sutton (2020)	NRES	FR‐56628 (C2254)	5 × 10^7^ TCID_50_ (IN)	Fever: 4/4 nasal shedding: 4/4, lethargy and tail hypotonia: 4/4	Viremia (qpcr): 4/4, mean peak viremia at 9 dpi (n = 4): 5.6 ± 0.3 log_10_ copy numbers/mL.
Van de Walle (2009)	NRES	Ab4 (rNY03_N752)	1.5 × 10^7^ PFU (IN)	Fever: 3/3, neurologic signs: 0/3.	Mean viremia score (RK 13 cells) at 8 dpi: 1.6. Viral genome copies in cerebrospinal fluid (pcr) = 0 for all horses.
		Ab4 rNY03_D752 (ORF mutant)		Fever: 6/6, moderate to severe ataxia: 2/6.	Mean viremia score (RK 13 cells) at 5 dpi: 1.8. Viral genome copies in cerebrospinal fluid (pcr) for horses without neurologic signs = 0 (n = 2), 118, and 178. Viral genome copies in cerebrospinal fluid (pcr) for horses with neurologic signs = 0 and 178. 630 genome copies/mL.
Wilson (2019)	NRES	Ab4	5 × 10^7^ PFU (IN)	Fever: 8/8, neurologic signs (9 dpi): 3/8 including mild hindlimb weakness (n = 1) or reduced tail tone, hindlimb ataxia, recumbency resulting in euthanasia (n = 2).	Viremia (pcr): 8/8, Peak mean viremia at 6 dpi: 705 ± 760 Gb copy number/10^6^ copies of β‐Actin.
Wimer (2018)	RCT	Ab4	10^7^ PFU (IN)	Peak mean rectal temperature at 2.5 dpi = 39.5 ± 0.3°C. Nasal shedding: 5/5. Neurologic signs: 0/5.	Viremia (pcr): 5/5, peak mean viremia at 5 dpi: 32.3 ± 0.4 copy number/5 × 10^6^ PBMC (Ct).
		Ab4ΔORF1/71		Peak mean rectal temperature at 3.5 dpi = 38.8 ± 0.1°C. Nasal shedding: 1/5. Neurologic signs: 0/5.	Viremia (pcr):5/5, peak mean viremia at 6 dpi: 33.4 ± 0.5 copy number/5 × 10^6^ PBMC (Ct).
Zarski (2021)	NRES	Ab4	5 × 10^7^ PFU (IN)	Fever: 7/7, nasal shedding: 7/7, severe neurologic signs requiring euthanasia: 1/7.	Viremia (pcr): 7/7. Peak viremia at 7 dpi seen in horse developing neurologic signs: 120 EHV‐1 copy number per 500 ng template DNA. Duration of viremia in this horse = 1 d. Peak viremia at 5‐7 dpi seen in four horses without neurologic signs: 299‐951 EHV‐1 copy number per 500 ng template DNA. Duration of viremia in these horses = 1‐3 d. Remaining horses had lower peak values.

Abbreviations: Ct, cycle threshold; d, day; IN, intranasal; NRES, non‐randomized experimental study; pi, post‐infection; PBMC, peripheral blood mononuclear cell; pcr, polymerase chain reaction; qpcr, quantitative real time PCR; RCT, randomized controlled trial; RK, rabbit kidney.

Four observational studies were retrospective reports of naturally occurring outbreaks.^38^, ^39^, ^40^, ^41^ Pusterla and coworkers grouped PCR‐positive horses into the following groups: asymptomatic horses (n = 27) and horses with neurological signs (n = 6).^40^ They found viral loads in blood from asymptomatic horses was lower when compared with viral loads seen in neurologic horses. No statistical differences in viral loads in nasal secretions were found between neurologic horses and asymptomatic horses.

There were 8 experimental studies that examined viremia and abortion (Table 3).^3^, ^18^, ^21^, ^22^, ^42^, ^43^, ^44^, ^45^ Six studies used the Ab4 strain, whereas other studies used the following strains: Army 183, OH03, KyB, Piber 178/83, or V592. Data evaluating peak viremia were limited to a single study.^3^ The remaining studies reported either the incidence of viremia and/or the duration of viremia. There was a single incidence of abortion in 1 pregnant mare exposed to the OH03 strain.^3^ Peak levels of virus in blood occurred at 5 days post‐infection and were reported at 10^3^ EHV‐1 gB copies/10^6^ beta actin copies. The affected mare also had the longest duration of viremia (4 days). Six other mares exposed to OH03 did not abort and peak viremia occurred at 5 to 7 days after infection. Peak viremia levels ranged from 10^2.9^ to 10^3.6^ EHV‐1 gB copies/10^6^ beta actin copies. In the same study, Gardiner et al.^3^ also exposed a cohort of 7 pregnant mares to the Ab4 strain, and 5 mares aborted. Aborted fetuses had lesions in the liver, adrenal glands, and spleen. The 2 mares that delivered healthy foals showed the shortest duration of viremia. Mares that aborted had peak viremia recorded between 5 and 9 dpi, reaching 10^2.3^‐10^3.2^ EHV‐1 gB copies/10^6^ beta actin copies. Peak viremia levels seen in mares that did not abort occurred at 5 to 7 days after infection and were nearly identical to those seen in mares that aborted (10^2.1^‐10^2.3^ EHV‐1 gB copies/10^6^ beta actin copies). Aborted fetuses from dams exposed to either strain of virus had lesions in the liver, adrenal glands, and spleen.

**TABLE 3:** Summary of main findings from studies evaluating abortion following EHV‐1 exposure.

Study identifier	Study design	Virus	Dose (route)	Outcome incidence	Main findings: viremia
Burki (1990)	NRES	Piber 178/83	10^7^ TCID_50_ (IN)	Fever ≥ 38.6°C: 7/10, abortion: 5/10	Viremia (horse kidney cells): 5/5 (PF that aborted); 4/5 (PF that delivered foals). SN titers (Δ 14 dpi − pre): PF that aborted: 268 ± 152; PF that delivered foals: 368 ± 140 (*P* = .36; post hoc analysis)
Gardiner (2012)	NRES	OHO3	5 × 10^7^ PFU (IN)	Fever ≥ 1°C rise above baseline: 7/7, abortion: 1/7	Viremia (qpcr): 7/7. Viremic between 5 and 8 dpi, with a duration of 1‐4 d. Mare #d that aborted was viremic for the longest duration of 4 d. Mare that aborted: Peak viremia (5 dpi): 10^3.0^ EHV‐1 gB copies/10^6^ beta Actin copies. Peak viremia in mares that did not abort at 5‐7 dpi: 10^2.9^‐10^3.6^ EHV‐1 gB copies/10^6^ beta Actin copies. Aborted fetuses had lesions in the liver, adrenal glands, and spleen.
		Ab4		Fever ≥ 1°C rise above baseline: 7/7, abortion: 5/7	Viremia (qpcr): 7/7. Viremic between 5 and 8 dpi with a duration of 1‐3 d. The two mares that delivered healthy foals showed the shortest duration of viremia. Mares that aborted: Peak viremia (5‐9 dpi): 10^2.3^‐10^3.2^. Peak viremia in mares that did not abort at 5‐7 dpi: 10^2.1^‐10^2.3^ EHV‐1 gB copies/10^6^ beta Actin copies. Aborted fetuses had lesions in the liver, adrenal glands, and spleen.
Gleeson and Coggins (1980)	NRES	Army 183	5 × 10^7.5^ TCID_50_ (nasal nebulized)	Fever: 11/11, abortion: 1/11. Nasal shedding 8/8.	Viremia (horse kidney cells): 10/11. Aborted foal was negative for virus. Mean duration of viremia 4.2 ± 2.4 d.
		KyB	5 × 10^6.5^ TCID_50_ (IN)	Fever: 5/10, abortion: 1/10. Nasal shedding 5/10.	Viremia (horse kidney cells): 8/10, mean duration of viremia 3.5 ± 2.9 d. Aborted foal was positive for virus.
Heldens (2001)	NRES	Ab4 (Controls)	2 × 10^6.0^ TCID_50_	Fever (>38.8°C): 4/5; abortion: controls 5/5.	Viremia (horse kidney cells): 5/5. Viral titers (PBMC) not provided. Mean duration of viraemia: 4.3 d.
		Ab4 (Vaccinated)		Fever (>38.8°C): 2/4, abortion: 1/5.	Viremia (horse kidney cells): 5/5. Viral titers (PBMC) not provided. Mean duration of viremia: 4.0 d.
Kydd (2003)	NRES	Ab4/8 (Controls)	1 × 10^5.0^ TCID_50_ (IN)	Fever: 9/9; nasal shedding: 3.6 ± 1.1, abortion: 9/9	Viremia: controls 9/9; duration of viremia: 4.3 ± 1.9 d. Viral titers (PBMC) not provided.
		Ab4/8 (Vaccinated)		Fever: 5/5, nasal shedding: 2.2 ± 1.1 d, abortion: 1/5.	Viremia: 5/5, duration of viremia: 4.0 ± 1.2 d. Viral titers (PBMC) not provided.
Mumford (1991)	NRES	AB4 Controls	10^7^ TCID_50_ (IN)	Fever: 9/9, abortion 8/9	Viremia (rabbit kidney cells): 9/9. Viral titers (PBMC) not provided.
		AB4 Vaccinated		Fever 10/10, abortion: 9/10	Viremia (rabbit kidney cells): 10/10. Viral titers (PBMC) not provided.
Mumford (1994)	NRES	V592	30 or 50 × 10^7.5^ TCID_50_ (IN aerosol)	Fever 8/10, abortion 1/10	Viremia (rabbit kidney cells): 10/10. Viral titers (PBMC) not provided.
		Ab4	20 × 10^6.7^ TCID_50_ (IN aerosol)	Fever 8/8, abortion 4/8	Viremia (rabbit kidney cells): 8/18, mean duration of viremia: 3.1 d. Viral titers (PBMC) not provided.
			30 × 10^7.5^ TCID_50_ (IN aerosol)	Fever 7/7, abortion 3/5	Viremia (rabbit kidney cells): 7/7, mean duration of viremia: 8.2 d. Viral titers (PBMC) not provided.
			10^3^ to 10^7^ TCID_50_ (IN)	Fever 35/41, abortion 27/35	Viremia (rabbit kidney cells): 15/15. Viral titers (PBMC) not provided.
Patel (2003)	NRES	Ab4 Control	2 × 10^5.7^ TCID_50_ (IN)	Fever: 6/6, nasal shedding 6/6, duration nasal shedding 6.5 ± 0.8 d, abortion 6/6	Viremia: 6/6, duration 2.3 ± 0.5 d. Viral titers (PBMC) not provided.
		Ab4 Vaccinated 4 mo		Fever: 3/5, nasal shedding 5/6, duration nasal shedding 2.2 ± 1.1 d, abortion 1/5	Viremia: 2/5, duration 2.0 ± 1.4 d. Viral titers (PBMC) not provided.
		Ab4 Vaccinated 5 mo		Fever: 2/6, nasal shedding 4/6, duration nasal shedding 2.5 ± 1.3 d, still birth 1/6	Viremia: 3/6, duration 1.0 ± 0 d. Viral titers (PBMC) not provided.

Abbreviations: Ct, cycle threshold; IN, intranasal; NRES, non‐randomized experimental study; PBMC, peripheral blood mononuclear cell; pcr, polymerase chain reaction; qpcr, quantitative real time PCR; RCT, randomized controlled trial; RK, rabbit kidney.

### 3.3 Quality assessment and risk of bias

**FIGURE 2:**
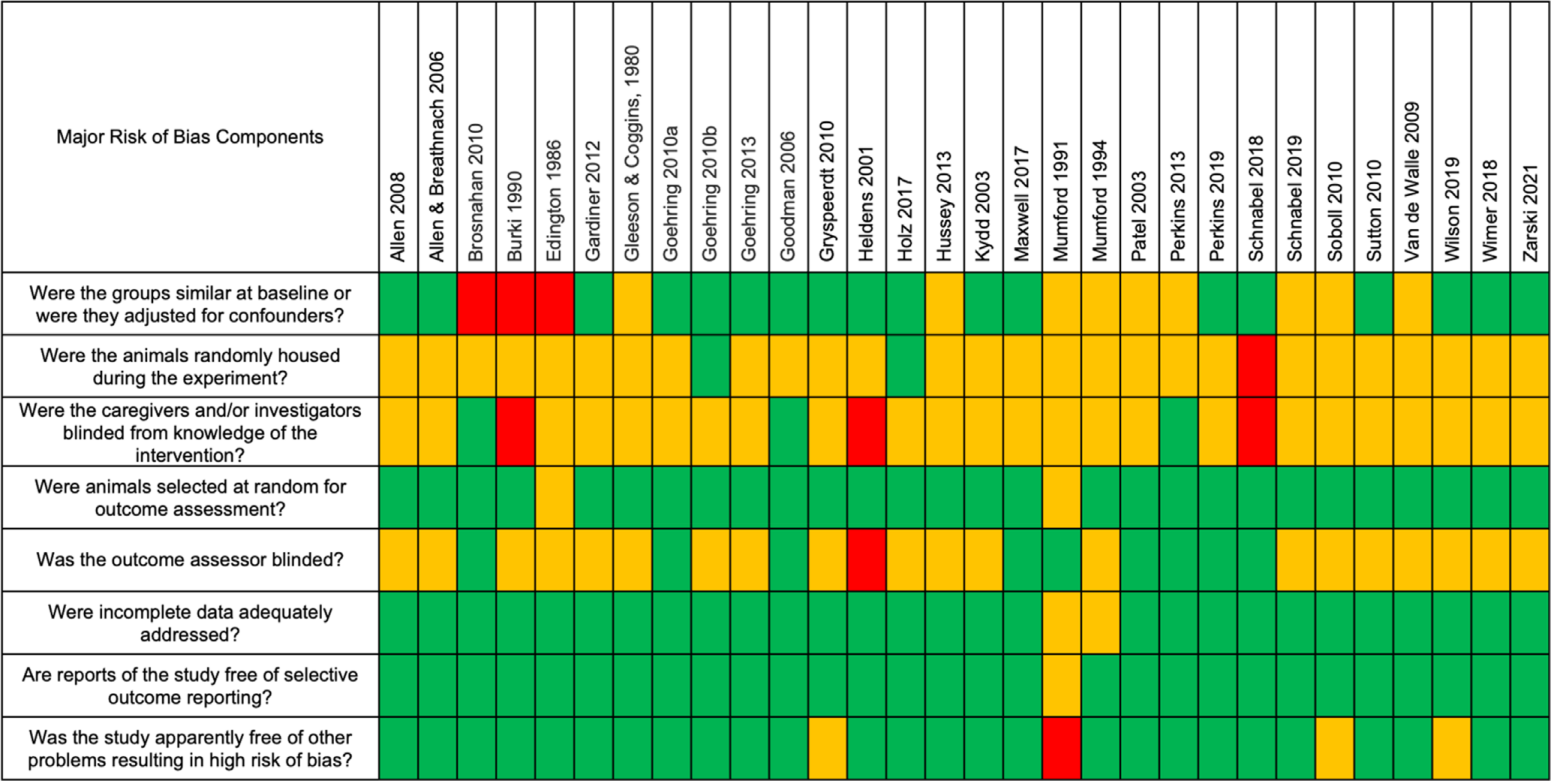
Risk of bias table of the included experimental studies. Green, yellow, and red denotes low, unclear, and high risk of bias, respectively.

The risk of bias evaluations for experimental studies are presented in Figure 2. Overall, the risk of bias was often unclear or unreported in many studies for the following 3 domains: were the animals randomly housed during the experiment; were the caregivers and/or investigators blinded from knowledge of the intervention; and was the outcome assessor blinded. In many experimental studies, the investigators were either aware of the treatment groups or could become aware of the treatment because animals were housed or pastured in different areas. A lack of blinding may introduce bias for the assessment of neurologic effects. This was thought less important for studies evaluating abortion. Some included experimental studies evaluated viremia in horses with prior vaccination or other treatments. The impact of these treatments on the outcomes was often considered unclear. Risk of bias was mostly low in other domains.

**FIGURE 3:**
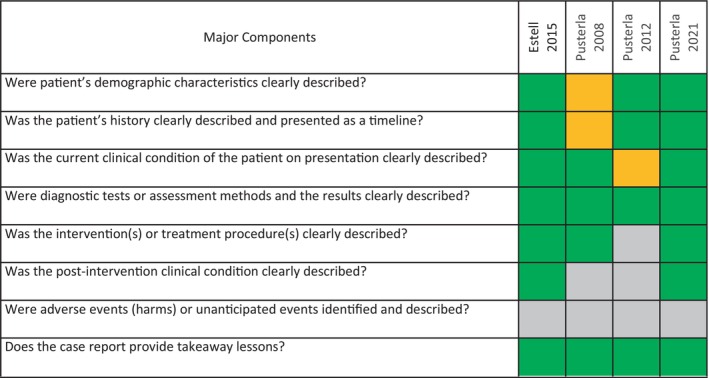
Study quality of the included observational studies. Green and orange denotes yes (clearly described) and unclear, respectively. Gray indicates the item was not relevant for the individual included study.

The quality of observational studies was evaluated using a separate tool developed for case reports. The result of this evaluation is provided in Figure 3. In general, the included case reports were deemed to be of high quality.

## 4 DISCUSSION

Our systematic review evaluated the association between either the level of viremia or duration of viremia and either EHM or abortion in horses infected with EHV‐1. Our results were mixed, and we did not include a meta‐analysis of the viremia data. The decision to not perform a quantitative analysis was driven by several factors, including the heterogeneity of the studies with respect to virus strains used, dose of virus used for challenge infection, different analytical methods (eg, PCR, plaque assays), insufficient reporting, low statistical power of the studies, among other factors. Additionally, there was variability in data collection and reporting over the timespan collected (eg, cycle thresholds, gene copies, TCID_50_, interval and duration of sampling). Another study limitation was the lack of data for horses that developed ataxia and other signs of neurologic disease vs those that did not. This often did not allow for meaningful statistical analysis and this problem will likely persist unless better models to study and experimentally induce EHM and EHV‐1 abortions are consistently used.

Results for EHM were variable. In 2008, Allen^8^ reported an association between the peak amount of viremia and the development of signs of neurologic disease in horses inoculated with the OH03 strain. Goodman et al.^32^ found a significant association overall between qPCR‐detected viremia following exposure to the OH03 strain and signs of neurologic disease, when treated as a categorical variable (*P* = .01 by Fisher's exact test). Maxwell and coworkers, who examined the effectiveness of valacyclovir either prophylactically or after the onset of fever in horses inoculated with the OH03 strain, provided some evidence in support of a possible association between higher levels of viremia and an increased risk of ataxia.^33^ One retrospective study reported that viral loads in blood were significantly different between the 2 groups with viremic neurologic horses having higher viral loads vs viremic asymptomatic horses.^40^ In contrast, Allen and Breathnach^30^ reported that the magnitude of viremia in 2 foals inoculated with a neuropathogenic strain of EHV‐1 were not significantly greater than in foals infected with paralytic strains of EHV‐1 that failed to exhibit neurological signs. Brosnahan et al.^15^ reported no significant difference in peak viremia between neurological and non‐neurological horses exposed to the Ab4 strain (*P* = .19). Holz and coworkers^19^ were unable to find a positive correlation between the duration of viremia and incidence of EHM following inoculation with different Ab4 mutant viruses, but reported that the onset of viremia and peak viremia levels correlated with fever responses seen in the horses. Perkins et al.^24^ reported no significant difference in either the onset of viremia, duration of viremia, or maximum viremia between horses that did or did not develop signs of neurologic disease following inoculation with the Ab4 strain. Wilson et al.^27^ reported no significant difference in viremia between horses exhibiting signs of neurologic disease and horses without neurologic signs following inoculation with the Ab4 strain. When comparing (for all horses) the data of viremia with those of neurological signs, the association (*P* = .01) suggested that the duration of viremia is more important for the risk of ataxia than the number of infected lymphocytes.^27^ However, a challenge with many of the conclusions drawn by the authors of the identified studies is, that the raw data of individual horses supporting these analyses are often inaccessible.

Our systematic review identified 8 experimental studies that examined viremia in pregnant horses following EHV‐1 exposure. Data evaluating peak viremia were limited to a single study.^3^ The remaining studies reported either the incidence of viremia and/or the duration of viremia. Viral titers were not accessible for most of these studies,^18^, ^21^, ^22^, ^42^, ^43^, ^44^, ^45^ thus no conclusions regarding an association between peak viremia and abortion following EHV‐1 exposure can be drawn. Data evaluating duration of viremia in these studies are also limited. As was the case with studies evaluating viremia and EHM, individual data are generally lacking in the studies evaluating viremia and abortion in EHV‐1 infected horses.

In conclusion, we found that there is convincing evidence in the literature that viremia is a pre‐requisite for occurrence of abortions and neurological disease. However, we were not able to find conclusive evidence for the role that duration or magnitude of viremia plays for the incidence of EHM or EHV‐1 abortions. This is not to say that such an association does not exist, but to firmly draw that conclusion and evaluate data in a systematic review, one cannot overstate the importance of consistent experimental study design including power and effects analysis as well as consistency in measuring and analyzing outcomes to be examined between research groups. Furthermore, it will be critical to develop and use models of EHM and EHV‐1 abortion that will reliably induce the desired outcome to answer these questions as well as test effectiveness of future therapeutic and preventative measures.

## CONFLICT OF INTEREST DECLARATION

Authors declare no conflict of interest.

## OFF‐LABEL ANTIMICROBIAL DECLARATION

Authors declare no off‐label use of antimicrobials.

## INSTITUTIONAL ANIMAL CARE AND USE COMMITTEE (IACUC) OR OTHER APPROVAL DECLARATION

Authors declare no IACUC or other approval was needed.

## HUMAN ETHICS APPROVAL DECLARATION

Authors declare human ethics approval was not needed for this study.

## Supporting information


**Data S1:** Supplementary Information.
