# Supplementary material for: Relationship between equine herpesvirus‐1 viremia and abortion or equine herpesvirus myeloencephalopathy in domesticated horses: A systematic review
Source: J Vet Intern Med. 2023 Dec 9;38(3):1872–91. doi: 10.1111/jvim.16948 (PMC11099755; doi:10.1111/jvim.16948)
Supplement: Supplementary file 1 — Data S1: Supplementary Information. [file JVIM-38-1872-s001.pdf]

Supplementary Materials Table 1

Complete Study Protocol:

Association Between Equine Herpesvirus-1 (EHV-1) Viremia and Either Abortion or Equine Herpesvirus Myeloencephalopathy (EHM) in Domesticated Horses: A Systematic Review

Research question: Is there an association between the level and duration of equine herpesvirus-1 viremia and either abortion or Equine Herpesvirus Myeloencephalopathy (EHM) in domesticated horses?

Problem formulation: The review question was developed and refined through a series of problem formulation steps including preliminary literature searches.

Searches: The review team will consider using existing systematic reviews to address or help to address its research question. English-language systematic reviews conducted within the last 5 years will be sought using searches in PubMed, PROSPERO (CRD), and CAMARADES.
A search for bibliographic references will be performed with the assistance of an experienced librarian through MEDLINE (via PubMed), LILACS (via Virtual Health Library), Cochrane Library (via Virtual Health Library) and EMBASE to locate studies. The search will be limited to domesticated horses and performed without sex, age, breed, or language restrictions. Only peer-reviewed publications will be considered. The search strategies will include the search for descriptors or words in the text related to abortion and viremia. The search will be developed with input from a librarian with expertise in the conduct of systematic reviews (See Appendix 1).

Types of studies to be included: Randomized controlled trials and observational studies.

Condition or domain being studied: Equine herpesvirus-1 (EHV-1) is a highly prevalent Alphaherpesviridae virus that infects horses worldwide. This respiratory virus is transmitted via direct horse-to-horse contact with contaminated nasal secretion as well as indirectly from contact with contaminated aborted fetuses, placenta, and fomites. This virus is associated with EHM, respiratory disease, abortion, neonatal death, and a neurologic disease known as Equine Herpesvirus Myeloencephalopathy (EHM). This review focuses on the associations between viremia and abortion and viremia and EHM. EHV-1 induced abortion often occurs in the third trimester. Some foals born to EHV-1 infected mares can be affected and may require euthanasia within days of birth. Clinical signs associated with EHM include hind-end weakness and incoordination (ataxia), urine dribbling or not able to urinate, loss of tail muscle tone, recumbent and unable to stand, depression. Secondary effects can include fever.

PICO Statement

PICO:

- **Population**: Domesticated equids without sex, age, or breed restrictions
- **Intervention / Exposure:** Equids experimentally infected or naturally exposed to EHV-1 infection.
- **Comparator:** Measurement/detection of viremia and association with severity of clinical, clinico-pathological and pathological signs of abortion, neonatal loss, or neurologic disease (Equine Herpesvirus Myeloencephalopathy -EHM).
- **Outcome:** All clinical outcomes that reflect symptomatic EHV-1 infection in horses with abortion, neonatal loss or EHM. Presence and degree of viremia.

Inclusion and Exclusion Criteria

Inclusion:

- Domesticated equids without age, breed, or immunological status restriction
- Any experimental challenge or natural infection with measurement of disease and of viremia.
- Study included clinical outcomes that reflect symptomatic EHV-1 infection resulting in either abortion, neonatal loss or EHM.
- Studies will not be excluded on the basis of year, language, or quality

Exclusion:

- Absence of an EHV-1 challenge trial or exposure
- Absence of the selected clinical or virological outcomes
- Wrong species of virus
- Wrong species (not equid)
- Purely descriptive observational studies
- No original data

Reason for exclusion for all studies will be recorded.

Main outcomes:

- Abortion or neonatal (week one) loss
- Neurologic signs suggestive of Equine Herpes Myeloencephalopathy

Secondary outcomes:

- Rhinopneumonitis: pyrexia with respiratory signs, including oculo-nasal discharge, elevated respiratory rate, cough, lethargy

Review team: The review team will include *redacted for review*. The review team will also be assisted by: *redacted for review*. If a member of the review team was a coauthor of a study under review, that member will recuse himself or herself from the evaluation of the quality of that study. The review team will be responsible for performing all aspects of the review, including conducting the literature searches; applying inclusion/exclusion criteria to screen studies; extracting data; assessing risk of bias for included studies; and analyzing and synthesizing data. The roles and responsibilities of the team members will be documented throughout the protocol. Throughout the course of its work, the review team will also engage others as needed. The involvement of those individuals will be documented.

Study selection

The evaluation of titles, abstracts, and the full text will be independently performed by teams of two reviewers (*redacted for review*); disagreements will be resolved by a third reviewer. A database management system (Covidence) will be used to manage and document these decisions.

Data extraction: Data will be collected and recorded (i.e., extracted) from included studies by one member of the review team (*redacted for review*) and checked by a second member for completeness and accuracy *redacted for review*). Any discrepancies in data extraction will be resolved through discussion. The extracted data will be used to summarize study designs and findings and/or to conduct statistical analyses.

Specific study endpoints to be extracted include:

- Demographic data:
    - Age, months of gestation, breed of horses
- Challenge protocol:

  - Virus strain
  - Virus dose, frequency, and route of administration
- Clinical signs (Yes/No, duration in days, severity):
  - Abortion and those associated with early neonatal loss
  - Neurologic disease, for example: ataxia; weakness; and urinary incontinence
  - Fever/rectal temperature
- Clinical Pathology
  - Leukogram changes or blood changes indicative of inflammatory disease
  - Cerebrospinal fluid color, cytology, and protein concentration
- Pathology
  - Reproductive tract or foal pathology
  - Nervous system pathology
- Virology (Yes/No, duration in days, quantitation)
  - Viral culture from blood and methods used
  - Viral DNA in blood and method used
  - Viral DNA in placenta and other tissues

Risk of bias evaluation: The risk of bias domains and questions for assessing risk of bias are based on established guidance for animal studies.[1] The SYRCLE risk of bias tool includes a common set of questions that are answered based on the specific details of individual studies to develop risk of bias ratings (using the following three options: low risk of bias; unknown risk of bias; or high risk of bias). Information or study procedures that were not reported are assumed not to have been conducted, resulting in an assessment of “unknown” risk of bias. Studies will be independently assessed by two assessors (to be determined) who answer all applicable risk of bias questions with one of three options following prespecified criteria. Risk of bias will be assessed at the outcome level. After assessors have independently made risk of bias

determinations for a study across all risk of bias questions, the two assessors will compare their results to identify discrepancies and attempt to resolve them. Any remaining discrepancies will be considered and resolved with the review team. The final risk of bias rating for each question will be recorded along with a statement of the basis for that rating. All risk of bias assessments will be recorded using Covidence. The following domains will be assessed:

- Blinding of participants and personnel:
    - Low Risk of Bias: Investigators **explicitly mention** that researchers were blinded AND it was unlikely that their blinding could have been broken AND housing conditions of the animals during the experiment are randomized to prevent unblinding AND timing of interventions were similar. Method used to blind personnel does not need to be explicitly stated.
    - Unclear or Not Reported: Blinding of investigators OR timing of interventions OR housing conditions are not explicitly reported.
    - High Risk of Bias: The authors explicitly mention that housing of experimental groups was not random (e.g., control horses were maintained at a different pasture or facility) OR timing of interventions differed between groups OR visible differences between control and experimental groups are anticipated to occur OR experimental methods differed between the groups.
- Random selection of animals for outcome assessment:
    - Low Risk of Bias: Investigators explicitly mention that researchers randomly picked animals for ALL outcome assessments. Risk of bias will be considered Low when all animals in all treatment groups are assessed for all outcomes of interest. Method of randomization does not need to be explicitly stated.
    - Unclear or Not Reported: Methods used to select animals for one or more outcome assessments are not provided OR it is not explicitly stated that all animals were assessed for all relevant outcomes.

- High Risk of Bias: The authors explicitly mention that only a subset of animals were used for one or more outcome assessments without mention of using a random method for selection.

- Blinding of outcome assessment
  - Low Risk of Bias: Investigators explicitly mention that researchers performing an outcome assessment were blinded AND it was unlikely that their blinding could have been broken AND housing conditions of the animals during the experiment are randomized to prevent unblinding AND timing of interventions were similar OR the outcome assessor not blinded, but the outcome is not likely to be influenced by a lack of blinding. Method used to blind personnel does not need to be explicitly stated.
  - Unclear or Not Reported: Blinding of investigators OR timing of interventions OR housing conditions are not explicitly reported.
  - High Risk of Bias: The authors explicitly mention that blinding was not used or blinding could likely be broken by the person assessing an outcome.
- Incomplete outcome data
  - Low Risk of Bias: Investigators explicitly present data indicating outcome data were available for all animals in a group OR missing outcome data is unlikely to be related to true outcome (e.g., technical errors, lost samples).
  - Unclear or Not Reported: It is unclear whether all outcome data are reported OR investigators state 'data not reported' for one or more outcomes of interest.
  - High Risk of Bias: The authors explicitly mention that some data is missing for one or more outcomes of interest AND a suitable explanation for missing outcome data is not provided.
  - Figures and Tables do not need to explicitly mention group sizes to confirm that all data are provided – this means we assume complete outcome reporting unless

otherwise stated. Lost data due to technical errors (or similar) does NOT result in a high risk of bias.

- Selective reporting
    - Low Risk of Bias: Investigators explicitly present data for all of the study's primary and secondary outcomes of interest.
    - Unclear or Not Reported: It is unclear whether all outcome data are reported.
    - High Risk of Bias: The methods and results sections indicate that one or more outcomes of interest were unreported OR investigators state 'data not reported' for one or more outcomes OR outcomes have been reported using measurements, analysis methods or data subsets (e.g., subscales) that were not pre-specified in the methods OR some reported outcomes were not pre-specified in the methods OR the study report fails to include results for a key outcome that would be expected to have been reported for such a study.
- Other bias
    - Low Risk of Bias: The study is free of other problems.
    - Unclear or Not Reported: There is insufficient information provided about statistical methods or the role of funders to evaluate risk of bias.
    - High Risk of Bias: The study may have been influenced by the funder OR the study had errors in statistical analyses (e.g., inappropriate pooling of data) OR design-specific risks of bias were present OR new animals were added to one or more groups to replace drop-outs from the original population OR some animals received additional treatment or drugs which might influence or bias results.

Strategy of data synthesis: A narrative synthesis (e.g., study design, year of publication, subject baseline demographics, sample size, country where study was conducted, interventions, and the results from each study) will be performed for each intervention. If we identify a

sufficient number of studies with adequate homogeneity, a meta-analysis will also be considered.

Confidence rating: Assessment of the body of evidence: The quality of evidence for each therapy will be evaluated using the GRADE system for rating the confidence in the body of evidence.[2,3] In brief, available studies on a particular outcome and virus will be initially grouped by key study-design features, and each grouping of studies is given an initial confidence rating by those features. The initial rating is downgraded for factors that decrease confidence in the results, including risk of bias, unexplained inconsistency, indirectness or lack of applicability, imprecision, or publication bias. The initial rating is upgraded for factors that increase confidence in the results, including large magnitude of effect, dose response, consistency across study designs/populations/animal models or species, consideration of residual confounding, other factors that increase confidence in the association or effect (e.g., particularly rare outcomes). Confidence ratings are independently assessed by members of the review team, and discrepancies will be resolved by consensus as needed. Confidence ratings will be summarized in evidence profile tables.

Protocol developed: 14 SEP 21

Search Strategy

PubMed

Concept 1: EHV1

"Herpesvirus 1, Equid"[Mesh] OR “equine herpesvirus 1”[tw] OR “equine herpes virus 1”[tw] OR “Equine abortion Virus”[tw] OR “Equine abortion Viruses”[tw] OR “EHV 1”[tw] OR EHV1[tw] OR “equid herpesvirus 1”[tw] OR "equid herpesvirus type 1"[tw] OR "equine herpesvirus type 1"[tw] OR "equine herpes virus type 1"[tw] OR "alphaherpesvirus"[tw]

Concept 2: Horses

horses[mesh] OR horse[tw] OR horses[tw] OR equid*[tw] OR equine*[tw] OR equus[tw]

Concept 3: Clinical outcomes

"Abortion, Veterinary"[Mesh] OR abortion[tw] OR "Stillbirth"[Mesh] OR "Stillbirth"[tw] OR "still birth"[tw] OR "neonatal loss"[tw] OR "neonatal death"[tw] OR "neurologic disease"[tw] OR "neurologic diseases"[tw] OR "Neurological disease"[tw] OR "central nervous system disease"[tw] OR "central nervous system diseases"[tw] OR "respiratory disease"[tw] OR "respiratory diseases"[tw] OR "elevated respiratory rate"[tw] OR "elevated respiratory rates"[tw] OR "Myeloencephalopathy"[tw] OR EHM[tw] OR "neuropathogenic strain"[tw] OR "neuropathogenic strains"[tw] OR "Fever"[Mesh] OR fever[tw] OR fevers[tw] OR pyrexias[tw] OR pyrexia[tw] OR Rhinopneumonitis[tw] OR "ocular disease"[tw] OR "ocular diseases"[tw] OR "Gait Ataxia"[Mesh] OR "Ataxia"[Mesh] OR ataxia[tw] OR ataxias[tw] OR ataxy[tw] OR incoordination[tw] OR coordination[tw] OR Dyscoordination[tw] OR "Rubral Tremors"[tw] OR "Rubral Tremor"[tw] OR Dyssynergia[tw] OR "Gait"[Mesh] OR gait[tw] OR "urine dribbling"[tw] OR "tail tone"[tw] OR weakness[tw] OR "Muscle Weakness"[Mesh]

Concept 4: Viremia

"Viremia"[Mesh] OR "Viremia"[tw] OR "Viremias"[tw] OR viraemia[tw] OR viraemias[tw] OR viremic[tw] OR viraemic[tw] OR "nasal shedding"[tw] OR ((nasal[tw] OR nasally[tw] OR nasopharyngeal[tw] OR Nasopharynx[tw]) AND (shed[tw] OR shedding[tw] OR secretion[tw] OR secretions[tw] OR discharge[tw]))

EHV-1_Web of Science

Concept 1: EHV1

TS=(“equine herpesvirus 1” OR “equine herpes virus 1” OR “Equine abortion Virus” OR “Equine abortion Viruses” OR “EHV 1” OR EHV1 OR “equid herpesvirus 1” OR "equid herpesvirus type 1" OR "equine herpesvirus type 1" OR "equine herpes virus type 1" OR "alphaherpesvirus")

Concept 2: Horses

TS=(horse OR horses OR equid OR equine OR equus)

Concept 3: Clinical Outcomes

TS=(abortion OR "Stillbirth" OR "still birth" OR "neonatal loss" OR "neonatal death" OR "neurologic disease" OR "neurologic diseases" OR "Neurological disease" OR "central nervous system disease" OR "central nervous system diseases" OR "respiratory disease" OR "respiratory diseases" OR "elevated respiratory rate" OR "elevated respiratory rates" OR "Myeloencephalopathy" OR EHM OR "neuropathogenic strain" OR "neuropathogenic strains" OR fever OR fevers OR pyrexias OR pyrexia OR Rhinopneumonitis OR "ocular disease" OR "ocular diseases" OR ataxia OR ataxias OR ataxy OR incoordination OR coordination OR Dyscoordination OR "Rubral Tremors" OR "Rubral Tremor" OR Dyssynergia OR gait OR "urine dribbling" OR "tail tone" OR weakness)

Concept 4: Viremia

TS=("Viremia" OR "Viremias" OR viraemia OR viraemias OR viremic OR viraemic OR "nasal shedding" OR ((nasal OR nasally OR nasopharyngeal OR Nasopharynx) AND (shed OR shedding OR secretion OR secretions OR discharge)))

Protocol Deviations:

Initial search completed September 14, 2021

References:

1. Hooijmans CR, Rovers MM, de Vries RB, et al. SYRCLE's risk of bias tool for animal studies. BMC Med Res Methodol 2014;14:43.

2. Guyatt GH, Oxman AD, Schunemann HJ, et al. GRADE guidelines: a new series of articles in the Journal of Clinical Epidemiology. J Clin Epidemiol 2011;64:380-382.

3. Guyatt GH, Oxman AD, Vist GE, et al. GRADE: an emerging consensus on rating quality of evidence and strength of recommendations. BMJ 2008;336:924-926.

9/16/21 9:37:22 AM

Supplementary Materials Table 2. Studies excluded during full text review. Reason for exclusion is also provided.

Study	Reason for exclusion
Allen GP, Kydd JH, Slater JD, Smith KC. 2004. Equid herpesvirus 1 and equid herpesvirus 4 infections. In: Infectious Diseases of Livestock 2nd Ed, Coetzer JAW and Tustin RC (Eds). Oxford, UK Oxford University Press. pp 829-859.	No original data
Attili AR, Colognato R, Preziuso S, et al. Evaluation of three different vaccination protocols against EHV1/EHV4 infection in mares: Double blind, randomized clinical trial. Vaccines (Basel). 2020;8(2):268.	Wrong outcomes
Bresgen C, Lämmer M, Wagner B, et al. Serological responses and clinical outcome after vaccination of mares and foals with equine herpesvirus type 1 and 4 (EHV-1 and EHV-4) vaccines. Vet Microbiol. 2012;160(1-2):9-16.	Wrong outcomes
Burgess BA, Tokateloff N, Manning S, et al. Nasal shedding of equine herpesvirus-1 from horses in an outbreak of equine herpes myeloencephalopathy in Western Canada. J Vet Intern Med. 2012;26(2):384-92.	Wrong outcomes
Burgess BA, Tokateloff N, Manning S, et al. Nasal shedding of equine herpesvirus-1 from horses in an outbreak of equine herpes myeloencephalopathy in Western Canada. J Vet Intern Med. 2012;26(2):384-92.	Duplicate
Bürki F, Rossmanith W, Nowotny N, et al. Viraemia and abortions are not prevented by two commercial equine herpesvirus-1 vaccines after experimental challenge of horses. Vet Q. 1990;12(2):80-6.	Duplicate
Crandell RA, Mock RE, Lock TF. Vaccination of pregnant ponies against equine rhinopneumonitis. Am J Vet Res. 1980;41(7):994-6.	Wrong outcomes
Dunowska M. How common is equine herpesvirus type 1 infection? Vet Rec. 2016;178(3):67-9.	No original data
Edington N, Bridges CG, Huckle A. Experimental reactivation of equid herpesvirus 1 (EHV 1) following the administration of corticosteroids. Equine Vet J. 1985;17(5):369-72.	Wrong outcomes
Garré B, Gryspeerdt A, Croubels S, et al. Evaluation of orally administered valacyclovir in experimentally EHV1-infected ponies. Vet Microbiol. 2009;135(3-4):214-21.	Wrong outcomes
Goehring LS, Landolt GA, Morley PS. Detection and management of an outbreak of equine herpesvirus type 1 infection and associated neurological disease in a veterinary teaching hospital. J Vet Intern Med. 2010;(5):1176-83.	Wrong outcomes
Goodman LB, Loregian A, Perkins GA, et al. A point mutation in a herpesvirus polymerase determines neuropathogenicity. PLoS Pathog. 2007;3(11):e160.	Wrong outcomes
Kydd JH, Hannant D, Robinson RS, Bryant N, Osterrieder N. Vaccination of foals with a modified live, equid herpesvirus-1 gM deletion mutant (RacHΔgM) confers partial protection against infection. Vaccine. 2020;38(2):388-398.	Wrong outcomes
Leutenegger CM, Madigan JE, Mapes S, et al. Detection of EHV-1 neuropathogenic strains using real-time PCR in the neural tissue of horses with myeloencephalopathy. Vet Rec. 2008;162(21):688-90.	Wrong outcomes

Matsumura T, Sugiura T, Imagawa H, et al. Epizootiological aspects of type 1 and type 4 equine herpesvirus infections among horse populations. J Vet Med Sci. 1992;54(2):207-11.	Wrong study design
McCartan CG, Russell MM, Wood JL, Mumford JA. Clinical, serological and virological characteristics of an outbreak of paresis and neonatal foal disease due to equine herpesvirus-1 on a stud farm. Vet Rec. 1995;136(1):7-12.	Wrong outcomes
Moreau P, Foursin M, Pronost S. Management of a focus of infection associated with equine herpesvirus 1 on a stud farm. Pratique Vétérinaire Equine 2012;44(173):31-36.	Wrong outcomes
Mori E, Lara Mdo C, Cunha EM, et al Molecular characterization of Brazilian equid herpesvirus type 1 strains based on neuropathogenicity markers. Braz J Microbiol. 2015;46(2):565-70.	Wrong outcomes
Mumford JA, Edington N. EHV1 and equine paresis. Vet Rec. 1980;106(12):277.	Wrong outcomes
Pusterla N, Mapes S, Akana N, et al. Prevalence factors associated with equine herpesvirus type 1 infection in equids with upper respiratory tract infection and/or acute onset of neurological signs from 2008 to 2014. Vet Rec. 2016;178(3):70.	Wrong outcomes
Pusterla N, Wilson WD, Mapes S, et al. Characterization of viral loads, strain and state of equine herpesvirus-1 using real-time PCR in horses following natural exposure at a racetrack in California. Vet J. 2009;179(2):230-9.	Wrong study design
Schröer U, Lange A, Glatzel P, et al. Die Bedeutung der Infektion mit dem equinen Herpesvirus Typ 1 (EHV-1) in einem deutschen Vollblutgestüt: Impfung, Abortgeschehen und Diagnostik [Relevance of infection with equine herpesvirus 1 (EHV-1) in a German thoroughbred stud: vaccination, abortion and diagnosis]. Berl Munch Tierarztl Wochenschr. 2000;113(2):53-9.	Wrong study design
Schröer U, Lange A, Glatzel P, et al. Die Bedeutung der Infektion mit dem equinen Herpesvirus Typ 1 (EHV-1) in einem deutschen Vollblutgestüt: Impfung, Abortgeschehen und Diagnostik [Relevance of infection with equine herpesvirus 1 (EHV-1) in a German thoroughbred stud: vaccination, abortion and diagnosis]. Berl Munch Tierarztl Wochenschr. 2000;113(2):53-9.	Duplicate
Smith KC, McGladdery AJ, Binns MM, Mumford JA. Use of transabdominal ultrasound-guided amniocentesis for detection of equid herpesvirus 1-induced fetal infection in utero. Am J Vet Res. 1997;58(9):997-1002.	Wrong outcomes
Smith KC, Whitwell KE, Binns MM, et al. Abortion of virologically negative foetuses following experimental challenge of pregnant pony mares with equid herpesvirus 1. Equine Vet J. 1992;24(4):256-9.	Wrong outcomes
Smith KC, Whitwell KE, Mumford JA, et al. Virulence of the V592 isolate of equid herpesvirus-1 in ponies. J Comp Pathol. 2000;122(4):288-97.	Wrong outcomes
Stasiak K, Dunowska M, Rola J. Outbreak of equid herpesvirus 1 abortions at the Arabian stud in Poland. BMC Vet Res. 2020;16(1):374.	Wrong outcomes
Studdert MJ, Hartley CA, Dynon K, et al. Outbreak of equine herpesvirus type 1 myeloencephalitis: new insights from virus identification by PCR and the application of an EHV-1-specific antibody detection ELISA. Vet Rec. 2003;153(14):417-23.	Wrong outcomes
Tsujimura K, Shiose T, Kokubun A, et al. A study on an inoculum dose of equine herpesvirus type 1 (EHV-1) mutant defective in the open reading frame of glycoprotein E and its vaccine effects. Utsunomiya, Japan Japanese Society of Equine Science (Nihon Uma Kagakukai) Equine Research Institute, Japan Racing Association 2004; 15:22.	Wrong outcomes

Tsujimura K, Shiose T, Yamanaka T, et al. Equine herpesvirus type 1 mutant defective in glycoprotein E gene as candidate vaccine strain. J Vet Med Sci. 2009;71(11):1439-48.	Wrong outcomes
Turan N, Yildirim F, Altan E, Sennazli G, Gurel A, Diallo I, Yilmaz H. Molecular and pathological investigations of EHV-1 and EHV-4 infections in horses in Turkey. Res Vet Sci. 2012;93(3):1504-7.	Wrong outcomes
Wilsterman S, Soboll-Hussey G, Lunn DP, et al. Equine herpesvirus-1 infected peripheral blood mononuclear cell subpopulations during viremia. Vet Microbiol. 2011;149(1-2):40-7.	Wrong outcomes
Yanni MI, Ebtsam AA, Ali HA, Hanna NM.Verification of molecular and conventional techniques used in the diagnosis of equine herpes virus in some Egyptian governorates. J Appl Vet Sci. 2021; 6(1):1-8.	Wrong outcomes
Yeo WM, Osterrieder N, Stokol T. Equine herpesvirus type 1 infection induces procoagulant activity in equine monocytes. Vet Res. 2013;44(1):16.	Wrong outcomes
